# Editorial: The atmospheric microbiota II: Microbial biomarkers and imprint of biological activity in the atmosphere

**DOI:** 10.3389/fmicb.2022.1055818

**Published:** 2022-10-18

**Authors:** Pierre Amato, Tina Šantl-Temkiv, Angelica Bianco

**Affiliations:** ^1^Université Clermont Auvergne, CNRS, INP Clermont Auvergne, Institut de Chimie de Clermont-Ferrand (ICCF), Clermont-Ferrand, France; ^2^Department of Biology, Aarhus University, Aarhus, Denmark; ^3^Stellar Astrophysics Centre, Department of Physics and Astronomy, Aarhus University, Aarhus, Denmark; ^4^Université Clermont Auvergne, CNRS, Laboratoire de Météorologie Physique (LaMP), Clermont-Ferrand, France

**Keywords:** atmosphere, biodiversity, microbial activity, long range transport, bioactive compounds

The atmosphere continuously carries billions of microbial cells that can reach distant locations by circulating with air currents. However, the natural aerial spread of microorganisms on a large scale, their genomic and phenotypic diversity, their functioning as living entities, and their impacts on atmospheric processes are still poorly understood. In this Research Topic, the authors have examined the great richness of airborne communities from Spain to Beijing, from Israel to Greenland, using culture-based or molecular approaches. They describe distant surface ecosystems interlinked with each other through the atmosphere by exchanges of microorganisms and molecular compounds, including the most extreme and remote environments such as polar regions (Gat et al.; Doting et al.; Jensen et al.). The biodiversity carried by airstreams is also explored as a reservoir of new biotechnologically and antimicrobially-relevant compounds (Sarmiento-Vizcaíno et al.; Yan et al.), and environmentally relevant structures such as the iconic bacterial ice nucleation protein described here in unprecedented molecular details (Hartmann et al.). Finally, the roles of oceans and seas in shaping the global atmospheric microbiome and its impacts on Earth's functioning are reviewed (Alsante et al.).

Gat et al. performed a size resolved investigation of bacterial and fungal biodiversity in aerosols in Israel, during clear days and during airborne dust episodes originating from the Sahara and the Arabian Desert. Microbial airborne communities occur as combinations of single, aggregated or attached cells. Their origin spans from local to distant sources and is a function of their size and thus their atmospheric residence time. Bacterial richness in the supermicron fraction of aerosol particles was shown to increase with increasing total particle number, i.e., during dust events. Microbial communities in the fine fraction of the particles (< 0.6 μm) were more similar to each other and less affected by sources, indicating higher spatial homogeneity. This work highlights the roles of sources and allometry in the aerial transport of microbes.

Meanwhile, Jensen et al. explored the Arctic aeromicrobiome in Northeast Greenland. Their observations also evidence the influence of distant sources of microorganisms on this pristine environment. Accordingly, marked seasonal variations could be observed in relation with the aerial connectivity of Arctic regions with temperate parts of the hemisphere during early spring, acting as sources of biogenic material. The influence of a snowstorm could be captured; this led to a strong washout of airborne bacteria, which resulted in concentrations dropping from 10^2^ to 10^5^ cells m^−3^ to undetectable levels, for several weeks following the event, due to very low local sources and absence of distant inputs during the Arctic late spring period. In this study Cyanobacteria were exhibiting elevated metabolic activity potential based on ribosome content, indicating their possible activity in the atmosphere and snow surface.

In contrast, Doting et al. studied post-depositional processes by quantifying the volatile organic compounds emitted from the Greenland ice sheet, including bare ice, cryoconite holes, and “red snow”, i.e., red algae blooms at the surface of snow. Their findings point to algae, fungi and Cyanobacteria thriving at the icy surface and serving as atmospheric sources of volatile organic compounds, released due to stress caused by high light radiation. Molecules such as alkanes, alkenes, aldehydes and N-containing VOCs were detected, some of which have known antifungal activity and could be utilized as defense strategies.

Alsante et al. propose a comprehensive review of the current knowledge in oceanic aerobiology. Whereas oceans and seas cover most of the Earth's surface, their associated airborne microbiota remain poorly characterized. Marine biogenic aerosols are generated by processes such as bubble-bursting, responsible for the aerosolization of microbes (viruses, archaea, bacteria and eukaryotes) from the water column, and in particular from the organic-rich surface microlayer (the thin interface between water and air). After emission, these can be dispersed over thousands of kilometers thus contributing, along with oceanic circulation, to biogeographical patterns at a global scale. In addition, biogenic marine aerosols can have influences on atmospheric processes and climate, notably by acting as cloud condensation and/or ice nucleating particles (CCN and/or IN).

Ice nucleation active (INA) proteins are probably among the most fascinating tools deployed by microorganisms for interfering with water aggregate states and therefore the water cycle. These proteins have a capacity to induce ice formation of supercooled water at higher sub-zero temperatures compared to natural abiotic particles and compounds. In addition to favoring the access to nutrients from plants in microbial phytopathogens by facilitating frost damages, these proteins also provide selective advantages to airborne microbes by shortening their atmospheric residence time, by inducing precipitation. Hartmann et al. studied the structure of ice nucleation proteins using a variety of modeling and experimental approaches. They reveal a novel beta-helix structure consisting of repeated stacks of beta strands, and demonstrate that dimers and higher-order oligomers of the protein drive its ice nucleation activity.

This Research Topic also provides support that the atmosphere serves as a reservoir of biodiversity and bioactive products. Sarmiento-Vizcaíno et al. isolated bacteria from precipitation in Spain capable of producing antibiotics. Chromatography and mass spectrometry allowed identifying products with antibiotic, antifungal, antitumoral, insecticidal, antiviral, cytotoxic and other properties. In the context of air quality and human health in urban environments, Yan et al. report variations in airborne microbiota composition linked with the presence of haze in Beijing, China. They also isolated antimicrobial-producing bacteria from airborne particulate matter, metabolites of which were active against human bacterial and fungal pathogens. These works highlight the potential benefits of exploiting airborne microbial diversity in the search for new bioactive molecules, as well as the dual beneficial and detrimental aspects of airborne microbiota for human health.

In summary, this Research Topic explores different facets of the fascinating field of aeromicrobiology, from long range transport of diverse microbes to potential biotechnologies, molecular characterization of potential biological adaptations to atmospheric dispersal, and to atmospheric processes. This continuation of a previous series of publications collected in 2018 in the Research Topic “The Atmospheric Microbiota” demonstrates the growing need for future research in atmospheric microbiology, and the associated grand challenges for the environment and health.

## Author contributions

All authors listed have made a substantial, direct, and intellectual contribution to the work and approved it for publication.

## Conflict of interest

The authors declare that the research was conducted in the absence of any commercial or financial relationships that could be construed as a potential conflict of interest.

## Publisher's note

All claims expressed in this article are solely those of the authors and do not necessarily represent those of their affiliated organizations, or those of the publisher, the editors and the reviewers. Any product that may be evaluated in this article, or claim that may be made by its manufacturer, is not guaranteed or endorsed by the publisher.

